# Clinical evaluation of dynamic [^18^F]FDG PET imaging to distinguish infection from inflammation in fracture-related infections

**DOI:** 10.1007/s00259-025-07563-x

**Published:** 2025-09-20

**Authors:** N. D. van Rijsewijk, M. Wouthuyzen-Bakker, J. H. van Snick, J. van Sluis, R. H.J.A. Slart, A. W.J.M. Glaudemans, F. F.A. IJpma

**Affiliations:** 1 Department of Nuclear Medicine and Molecular Imaging, University Medical Center Groningen, University of Groningen, Groningen, the Netherlands; 2 Department of Medical Microbiology and Infection Prevention, University of Groningen, University Medical Center Groningen, Groningen, the Netherlands; 3 Biomedical Photonic Imaging Group, Faculty of Science and Technology, University of Twente, Enschede, The Netherlands; 4 Department of Trauma Surgery, University of Groningen, University Medical Center Groningen, Groningen, the Netherlands

**Keywords:** Dynamic, PET/CT imaging, [^18^F]FDG, Infection, Inflammation, Fracture-related infection

## Abstract

**Background:**

Standard [^18^F]FDG PET/CT is widely used to detect infections, but is limited in differentiating bacterial infection from inflammation due to overlapping uptake patterns. This distinction is particularly challenging in fracture-related infection (FRI), due to multiple factors, including recent surgery, soft tissue injury, fracture healing, and surgical implants. Dynamic [^18^F]FDG imaging over time may overcome this limitation by capturing a rapid, sustained uptake seen in infections, driven by ongoing immune and microbial activity, as opposed to the potentially slower uptake in inflammation. This study investigates the potential of dynamic [^18^F]FDG PET/CT using time-activity curves (TACs) to distinguish infection from inflammation in patients with suspected FRI.

**Methods:**

A prospective study was performed in patients referred for [^18^F]FDG PET/CT examination to detect (the extent of) FRI from December 2021 until November 2024. Final clinical diagnosis of FRI was established according to the criteria defined by the FRI consensus group and used as a reference standard. Dynamic PET imaging was performed using a long axial field-of-view PET/CT system over a 65-minute period after [^18^F]FDG injection. TACs were analyzed to compare uptake patterns between confirmed infections and inflammation, and diagnostic accuracy was calculated and compared to conventional standard imaging.

**Results:**

Thirty-one patients with 33 fractures suspected of infection were included. Infection was clinically confirmed in 14 fractures (42%). Diagnosis of FRI based on dynamic imaging using TACs resulted in a high sensitivity of 86%, specificity of 100%, PPV of 100% and NPV of 90%, resulting in a diagnostic accuracy of 94%. In the infection group, Fracture SUV_peak_ increased progressively over time, while in the inflammation group, uptake appeared to reach a plateau *(p* < 0.001). Visual interpretation of the dynamic imaging yielded a diagnostic accuracy of 82%. For standard imaging, quantitative and visual assessments showed diagnostic accuracies of 91% and 88%, respectively.

**Conclusion:**

This proof-of-concept study demonstrates that dynamic [^18^F]FDG PET/CT using TACs can distinguish infection from inflammation in patients with suspected FRI, with high diagnostic accuracy observed, and suggesting a potential advantage over standard imaging. Larger-scale studies are needed to validate these results and explore the full clinical potential of dynamic imaging in infection management.

**Supplementary Information:**

The online version contains supplementary material available at 10.1007/s00259-025-07563-x.

## Introduction

The clinical use of standard [^18^F]FDG PET/CT for diagnosing infections has been well established over the years [1]. By visualizing areas with increased glucose metabolism due to inflammatory responses or microbial activity, this imaging modality can localize and assess the extent of suspected infections [2]. However, interpretation of a single time-point image can be challenging, especially in differentiating bacterial infections from inflammation. This difficulty arises in clinical scenarios involving soft tissue injury, bone fractures, recent surgeries, and/or the presence of osteosynthesis plates, screws or nails [3]. Relying solely on single time-point imaging may oversimplify the underlying biological complexity, resulting in potential diagnostic uncertainty.

Both infections and inflammation show increased [^18^F]FDG uptake [1, 3]. In infections, high glucose metabolism is caused by actively proliferating microorganisms and the immune response which includes the recruitment of neutrophils and macrophages. In inflammation, the [^18^F]FDG uptake is mainly caused by localized tissue repair and non-infectious immune responses [2]. In theory, [^18^F]FDG uptake over time may provide a critical distinction between the two processes. Due to the continuous metabolic activity of immune cells and microorganisms, it is assumed that infections show a rapid and sustained [^18^F]FDG uptake. In contrast, reactive processes and inflammation lack the metabolic activity associated with active microbial growth, and therefore potentially show a slower uptake rate [1, 3, 4]. Dynamic imaging and the insight into the temporal changes in [^18^F]FDG uptake may therefore be a more precise approach to differentiate between infection and inflammation rather than standard imaging alone.

Fracture-related infections (FRIs) are one of the complex scenarios in which such differentiation is particularly important. FRIs are characterized by a complex interplay with microorganisms, immune responses, tissue damage, bone healing, recent surgical interventions, and often the presence of metallic implants [5]. Distinguishing infection from inflammation therefore remains challenging, as both processes can exhibit overlapping clinical and imaging findings on standard scans. Furthermore, biofilm formation on implants can result in subtle and diffuse [^18^F]FDG uptake, further mimicking inflammatory conditions [6]. By assessing metabolic changes over time, dynamic imaging may offer insights into these metabolic variations, enabling more precise differentiation between infection and inflammatory processes.

The introduction of long axial field-of-view (LAFOV) PET/CT systems further enhances this approach by allowing simultaneous visualization of larger anatomical regions. This broader coverage facilitates dynamic imaging of long implants (often > 25 cm) and surrounding tissue in a single acquisition, improving practicality and workflow compared to standard field-of-view systems. With increased sensitivity, LAFOV PET/CT provides higher count statistics and reduced noise, resulting in improved image quality and contrast [7]. These improvements enhance the detectability of subtle infections, such as low-grade or biofilm-associated cases, which may present with mild, diffuse, and/or heterogeneous [^18^F]FDG uptake. Given the clinical importance of early and accurate diagnosis of FRI, including the urgency to avoid unnecessary implant exchange or prolonged antibiotic use, enhancing diagnostic accuracy is essential.

This prospective cohort study aims to evaluate the potential of dynamic [^18^F]FDG PET/CT imaging to distinguish infection from inflammation in patients with suspected FRI. In this study, the primary research question is to determine whether dynamic imaging can reliably differentiate infection from inflammation using time-activity curves (TACs).

## Materials and methods

### Study population

A prospective cohort study was performed in patients with a suspected FRI treated at a level 1 trauma center between December 2021 and November 2024. Patients were eligible for inclusion when they were referred to the nuclear medicine department for [^18^F]FDG PET/CT. The indication for nuclear imaging was to detect infections and, if present, to visualize the extent of the infection for surgical planning. Patients who suffered from claustrophobia, were unable to lie still for 1.5 h, or those with a suspected malignancy were excluded. The local Medical Ethical Commission approved this prospective study (METC number 2023/040) and informed consent was obtained.

The final clinical diagnosis of FRI was established based on the (refined) diagnostic criteria by the FRI consensus group [8, 9]. Confirmation of an FRI was obtained through clinical evaluation by observing a fistula, wound dehiscence exposing bone or implant, or presence of pus within the fracture site. Surgical confirmation of FRI involved the identification of microorganisms that were phenotypically indistinguishable, isolated from at least two deep tissue samples, or visible microorganisms on histological examination.

The following data were collected from the electronic medical record: patient demographics, clinical parameters, imaging details, and microbiological results. Clinical parameters included C-reactive protein (CRP), erythrocyte sedimentation rate (ESR) and leukocyte count (LC), with measurements within a three-week window to the PET/CT scan, but excluding post-operative results.

### PET/CT acquisition, image reconstruction and analysis

All scans were performed using a long axial field-of-view PET/CT system (106 cm, Biograph Vision Quadra; Siemens Healthineers, Knoxville, TN, USA). Dynamic PET imaging was performed in a single bed position and began immediately after intravenous [^18^F]FDG injection and lasted 65 min. In 2021 and 2022, patients received a standard intravenous administration of 3 MBq/kg[^18^F]FDG according to EANM guidelines [4]. Since 2023, the administered activity has been lowered to 2 MBq per kilogram body weight to minimize radiation exposure, in accordance with the ALARA (as low as reasonably achievable) principle. All patients were prepared with a fasting period of at least 6 h prior to the intravenous tracer injection.

Low dose CT was performed for localization and attenuation correction. Reconstruction used an iterative metal artifact reduction algorithm if applicable. Standard and dynamic attenuation corrected (AC) PET images were reconstructed using a point spread function (PSF) algorithm in 4 iterations and 5 subsets with application of time-of-flight (TOF) and a Gaussian filter of XYZ 5.00 mm, resulting in PET images with a matrix of 220 rows x 220 columns with a voxel size of 3.30 × 3.30 × 2.00 mm^3^. These reconstructions used a maximum ring difference (MRD) of 85 (acceptance angle of 18°). All standard images were reconstructed using European Association of Nuclear Medicine Research Ltd. (EARL) standard 2 settings (EARL2) [10]. The dynamic reconstructions were acquired in 31 frames in the following order: 6 × 10 s, 3 × 20 s, 6 × 30 s, 5 × 1 min, 11 × 5 min, using the same reconstruction parameters as for the standard EARL2 reconstruction. The final three minutes of the total scan duration were used to reconstruct the standard images, with and without attenuation correction.

Qualitative analysis was performed on both standard and dynamic images, by one radiology and nuclear medicine resident engaged in PET research (NR, over 4 years of experience) and one experienced nuclear medicine physician (AG, 17 years of experience). Disagreements were resolved in consensus, involving another nuclear medicine physician (RS, 24 years of experience). On the standard AC image, assessment was performed using the uptake pattern (heterogeneous, homogeneous/diffuse or focal) and intensity of [^18^F]FDG uptake (using a 5-point scale: 0 – no uptake, 1 – uptake comparable to contralateral side, 2 – increased uptake but less than blood pool, 3 – uptake similar to blood pool, 4 – uptake more than blood pool, 5 – intense uptake more than twice the blood pool) at the suspected infection site, the presence of [^18^F]FDG avid lymph nodes in the suspected drainage pathway and increased bone marrow and spleen uptake (similar to or more than liver uptake, if visualized). Scan quality was assessed using a 4-point scale: 0 – bad, 1 – intermediate, 2 – good and 3 – excellent, with a correction of minus 1 point for minor and minus 2 points for major increased muscle and soft tissue uptake or movement. Furthermore, persistence of the [^18^F]FDG avid infection or inflammation lesions was assessed on the non-attenuated corrected (NAC) images. The dynamic images were visually assessed using increased [^18^F]FDG uptake in the early phase (first 10 min post injection) compared to the contralateral side, persistent tracer accumulation and the change in tracer uptake during the whole acquisition at the suspected infection site.

Syngo.via VB80 software (Siemens Healthineers, Knoxville, TN, USA) was used for semiquantitative analysis. Ellipsoidal volumes of interest were drawn on the standard and dynamic images at the following locations (if applicable): suspected infection site, contralateral side and blood pool (descending aorta or femoral artery). Standardized uptake values (SUVs) were calculated and corrected for glucose. Specifically, SUV_max_ (the highest voxel value within the volume of interest), SUV_peak_ (the average SUV within a spherical volume of 1 cm^3^ region centred on the area of highest uptake), and SUV_mean_ (the average uptake within the entire volume of interest) were assessed. Time-activity curves (TACs) were generated based on the dynamic reconstruction.

### Statistical analysis

The primary outcome measure of this study is the diagnostic accuracy of using TACs of dynamic [^18^F]FDG PET/CT to diagnose infection. Secondary analyses included comparisons with the diagnostic performance of visual and quantitative standard imaging.

Descriptive statistics were used to summarize patient characteristics and quantitative assessment. Normality of continuous variables was assessed with the Shapiro-Wilk test, and equality of variances with Levene’s test. Depending on data distribution, group comparisons were performed using independent t-tests or Mann-Whitney U tests for continuous variables, and Chi-square or Fisher’s exact tests for categorical variables.

To calculate diagnostic accuracy of the visual assessment of the standard [^18^F]FDG PET/CT scan, patients were categorized as suspected infection when they met the following criteria: (1) an [^18^F]FDG uptake intensity > 3 at the suspected infection site, (2) Heterogenous or focal uptake at the fracture site, and (3) persistence of the [^18^F]FDG uptake on NAC images [1]. All others are classified as “suspected inflammation”. For standard quantitative analysis, classification was based on a cut-off for SUV_peak_ determined by ROC curve analysis, with values above the threshold being categorized as suspected infection.

For dynamic visual assessment, increasing accumulation of tracer activity over time was considered indicative of infection. For dynamic quantitative analysis, time-activity curves of SUV_peak_ were analysed using linear mixed-effects models. Fixed effects included group (infection vs. inflammation), time, and the group-by-time interaction, while random slopes for time accounted for inter-individual variation. To classify individual patients based on dynamic TACs, linear regression models were generated for each group (infection and inflammation) after t = 10 min. Each patient’s time-activity curve was compared to these models by calculating the mean squared error (MSE). Patients were categorized as suspected infection when the MSE of their TAC was closer to the infection regression model than to the inflammation model, and vice versa for suspected inflammation. All classification methods were evaluated against the clinical diagnosis as the reference standard. Table 1 summarizes the method-specific classification criteria and thefollowing definitions were applied:

- True positive (TP): Imaging suspected infection, and clinical diagnosis confirmed infection.
- False positive (FP): Imaging suspected infection, but clinical diagnosis did not confirm infection.
- True negative (TN): Imaging suspected inflammation, and clinical diagnosis confirmed no infection.
- False negative (FN): Imaging suspected inflammation, but clinical diagnosis confirmed infection.

Diagnostic performance was reported as sensitivity, specificity, positive predictive value (PPV), negative predictive value (NPV), and overall accuracy.

**Table 1:** Method-specific classification criteria for imaging outcome

Method	Classification as suspected infection	Classification as suspected inflammation
Standard PET – visual assessment	(1) Uptake intensity grade > 3 at suspected infection site, and (2) heterogeneous/focal uptake at fracture site, and (3) persistence on non-attenuated corrected image	Scans not fulfilling all three criteria for suspected infection
Standard PET – quantitative assessment	SUV_peak_ above the ROC-derived threshold	SUV_peak_ below the ROC-derived threshold
Dynamic PET – visual assessment	Visual increase of tracer accumulation over time	Visual decrease or equivocal tracer accumulation over time
Dynamic PET – quantitative assessment	Patient’s time-activity curve compared with group regression models (after t = 10 min) with their mean squared error closer to infection model	Patient’s TAC compared with group regression models (after t = 10 min) with their MSE closer to inflammation model

All results were considered statistically significant with a *p*-value < 0.05. Missing data were excluded pairwise. Statistical analyses were performed using IBM^®^ SPSS^®^ Statistics (Version 28; IBM Corp., Armonk, NY, USA) and Python (version 3.12) with the following packages: *matplotlib*, *numpy*, *pandas*, *scipy*,* seaborn*,* sklearn and statsmodels*.

## Results

### Patient and fracture demographics

This study included 31 patients (24 men, 7 women; mean age 52 years) with 33 fractures evaluated for suspected infection. Clinical confirmation of infection was obtained in 14 fractures (42%). Most suspected fracture infection sites were in the lower extremities (30/33, 91%), resulting in lower extremities-only scans in 28/33 (85%). Metallic implants were present in 26/33 (79%) cases. Clinical signs included a fistula in 10 cases (30%), wound breakdown in 2 (6%), and pus/debris in 7 fractures (21%).

Surgery was performed in 25/33 fractures after PET/CT imaging, with a median duration from PET/CT to surgery of 1.6 months [0.6–3.2]. A deep wound culture was taken intra-operatively in all 25 fractures and sonication of the osteosynthesis material was performed in 10 cases. In 14 patients, deep tissue microorganisms were identified that were phenotypically indistinguishable. The median time between the initial trauma and PET/CT scan was 16.9 months [9.9–62.4]. The time between the latest surgery and PET/CT imaging was 12.5 months [6.6–34.4]. The mean time of follow-up was 13.9 months (10.5–17.3).Tab. 2 

**Table 2:** Patient and fracture demographics. Data are presented as mean (95% CI) or as median [Q1-Q3]. Abbreviations: DWC = deep wound culture, OSM = osteosynthesis material

	All		Infection		Inflammation		*p*
Patient demographics	*N* = 31		*N* = 14		*N* = 19		
Age (years)	51.6	(45.6–57.5)	65.0	[50.3–72.5]	46.0	(39.6–52.4)	***0.017***
*Sex* Male Female	24 7	(77.4%) (22.6%)	9 5	(64.3%) (35.7%)	16 3	(84.2%) (15.8%)	*0.238*
CRP (mg/L)	10.0	[7.0–30.0]	23.5	(12.5–34.5)	10.0	[2.8–16.0]	*0.197*
Leukocyte count (x 10^9^/L)	8.6	(7.3–10.0)	8.2	(6.1–10.3)	9.7	(7.7–11.6)	*0.279*
ESR (mm/h)	40.0	(23.9–56.1)	55.7	(32.5–79.0)	21.9	(10.1–33.6)	***0.008***
Glucose (mmol/L)	5.3	[5.0–6.0]	5.7	(5.1–6.4)	5.3	(5.1–5.5)	*0.162*
Administered activity (MBq)	170	[150–213]	193	(155–231)	180	(157–202)	*0.492*
Fracture demographics	*N* = 33		*N* = 14		*N* = 19		
*Bone/Joint* Tibia Femur Ankle Fibula Humerus Metatarsal V Pelvic bones Ulna (olecranon)	15 9 4 1 1 1 1 1		5 5 1 1 1 0 0 1		10 4 3 0 0 1 1 0		*0.503*
*Scanned body part* Lower extremities Whole body Shoulder-to-knee Abdomen-to-mid-tibia	28 2 2 1		9 2 2 1		19 0 0 0		***0.008***
*Metallic Implants* *Yes* *No*	26 7		9 5		17 2		*0.106*
*Sinus tract* Yes No	10 23		10 4		0 19		***< 0.001***
*Wound breakdown* Yes No	2 31		2 12		0 19		*0.172*
*Pus in fracture* Yes No N/A	7 18 8		7 6 1		0 12 7		***0.002***
*Sample* Intra-operative DWC DWC + sonication OSM N/A	15 10 8		6 8 0		9 2 8		*0.099*
*Deep tissue microorganisms* *2x* Yes No N/A	14 11 8		14 0 0		0 11 8		***< 0.001***
*Histological visible organisms* Yes No N/A	0 6 27		0 4 10		0 2 17		
Time between trauma and PET (months)	16.9	[9.9–62.4]	11.2	[7.6–40.4]	31.5	[13.8–65.5]	***0.038***
Time between last surgery and PET (months)	12.5	[6.6–34.4]	7.7	[4.5–11.3]	15.1	[10.8–55.3]	***0.016***
Time between PET and new surgery (months)	(*N* = 25)		(*N* = 14)		(*N* = 11)		***0.008***
	1.6	[0.6–3.2]	1.0	[0.6–2.3]	2.2	[1.5–5.6]	
Follow-up time after PET (months)	13.9	(10.5–17.3)	15.6	(9.9–21.3)	12.6	(8.1–17.1)	*0.381*

### Visual analysis

**Fig. 1:**
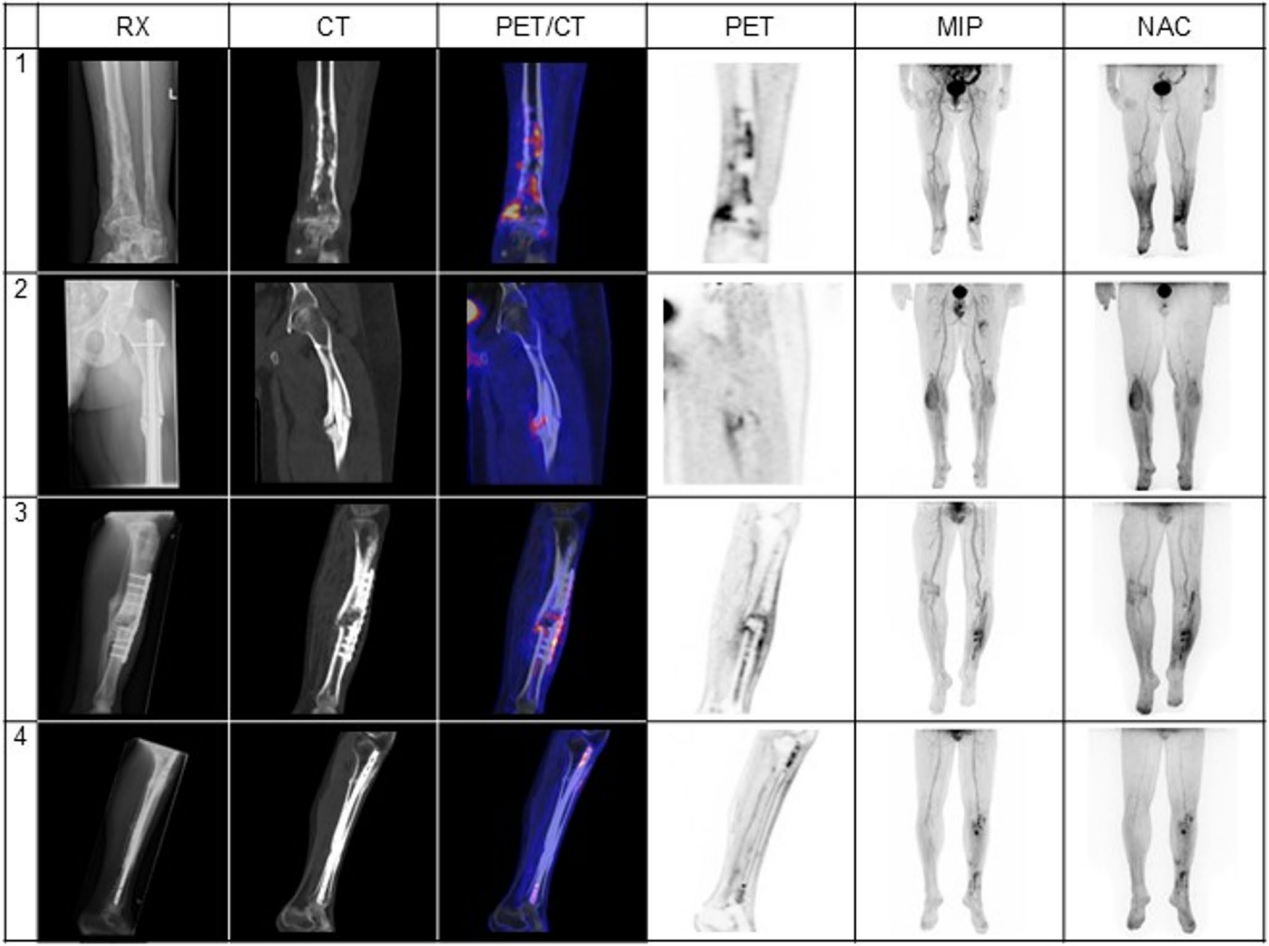
Assessment of standard [^18^F]FDG PET/CT imaging in patients with suspected fracture-related infections. Patient 1 (true positive scan): a confirmed infected non-union of the medial malleolus was classified as true positive as the scan shows intense focal [^18^F]FDG uptake with an SUV_peak_ of 6.65 around the medial malleolus of the left ankle, which persists at the NAC images. Patient 2 (true negative scan): inflammation in a femoral non-union shows a more focal pattern around the fracture site with grade 4, with an SUV_peak_ of 3.31. The [^18^F]FDG uptake at the suspected infection site is not persistent on the NAC image, classifying the scan as true negative. Patient 3 (false positive scan): a tibial non-union shows high focal and heterogeneous uptake at the fracture site and osteosynthesis material with an SUV_peak_ of 4.75, which is persistent at the NAC image, but no infection could be confirmed based on the criteria of the fracture-related infection consensus definition. Patient 4 (visual assessment: true positive scan, quantification: false negative scan): a tibial non-union shows high focal uptake around the threaded holes of the osteosynthesis material, more pronouncedly proximal with a SUV_peak_ of 3.86, which persist on the NAC images. This patient was correctly classified as infection by visual scan analysis, but falsely classified as negative by quantification (SUV_peak_ < 4.40). Abbreviations: RX = radiograph, CT = computed tomography, PET = positron emission tomography, MIP = maximum intensity projection, NAC = non-attenuated corrected, SUV = standardized uptake value

A summary of visual scan analysis can be found in Table 3. Uptake intensity was significantly higher in the infection group (*p* < 0.001), with all 14/14 scans showing grade 4 or 5 uptake. In contrast, inflammation cases (19/19) showed a wider distribution, with 17/19 scans (89%) between grades 0 and 3, and only 2/19 (11%) reaching grade 4 or 5. Uptake patterns also differed significantly (*p* = 0.026): focal and heterogeneous uptake patterns were observed in 14/14 (100%) of infection cases, while 9/19 (47%) of inflammation cases showed diffuse or non-increased uptake. FDG-avid lymph nodes were present in 13/14 (93%) infection cases versus 6/19 (32%) inflammation cases (*p* < 0.001). Persistent uptake on NAC images was seen in all infection cases (14/14, 100%) versus 11/19 inflammation cases (58%) (*p* = 0.010). An illustration of the assessment of standard [^18^F]FDG PET/CT imaging can be found in Fig. 1.

**Table 3:** Visual analysis of standard and dynamic [^18^F]FDG PET/CT scans in infection and inflammation

		Infection (*N* = 14)	Inflammation (*N* = 19)	*p*-value
Scan quality				*0.199*
	Bad Intermediate Good Excellent	1 5 3 5	0 5 10 4	
Standard scan				
Uptake intensity				***< 0.001***
	0 1 2 3 4 5	0 0 0 0 6 8	2 3 2 6 5 1	
Uptake pattern				
	Not increased Diffuse Focal Heterogeneous	0 0 5 9	3 6 3 7	
FDG avid lymph nodes				**< 0.001**
	No Yes N/A	1 13 0	12 6 1	
Bone marrow uptake				
	No Yes N/A	6 1 0	3 0 16	
Spleen uptake				
	No Yes N/A	5 0 9	0 0 19	
NAC persistent uptake				
	No Yes	0 14	8 11	
Dynamic scan				
Increased uptake in early phase				*0.101*
	No Yes N/A	1 11 2	8 11 0	
Increasing tracer accumulation				
	No Yes	0 14	13 6	

Diagnosis of fracture-related infection based on the visual analysis of the standard scan using the three criteria showed 14 true positives, 15 true negatives and 4 false positives compared to the clinical classification. There were no cases classified as false negative. This resulted in a sensitivity of 100%, specificity of 78.9%, PPV of 77.8%, NPV of 100% and a diagnostic accuracy of 87.9%.

In dynamic imaging, early-phase increased uptake did not significantly differ (*p* = 0.101). However, increasing tracer accumulation over time was observed in all infection cases (14/14, 100%) and only 6/19 inflammation cases (32%), with a significant difference (*p* < 0.001). This method yielded 14 TP, 13 TN, and 6 FP, resulting in a sensitivity of 100%, specificity 68.4%, PPV 70.0%, NPV 100%, and accuracy 81.8%.

## Quantitative assessment

### Standard scan

Comparison between the infection and inflammation group showed significant differences in fracture SUV_peak_ (*p* < 0.001), with a median (95% CI) of 6.97 (5.50–8.44) and 2.58 (1.93–3.22) for the infection and inflammation group, respectively. Significant differences were also observed in fracture SUV_max_ (*p* < 0.001) and SUV_peak_ compared to the contralateral side (*p* = 0.008), with higher SUV measurements for the infection group. There was no significant difference in blood pool activity between the two groups in femoral artery SUV_mean_. An overview of all SUV measurements and their ratios from the standard [^18^F]FDG PET/CT imaging are summarized in Supplementary Tables 1 and visualized in Supplementary Fig. 1.

Fracture SUV_peak_ demonstrated the highest diagnostic performance by ROC-curve analysis. Using an optimal cut-off of 4.40 SUV, the area under the curve (AUC) was 0.962, yielding a sensitivity of 85.7%, specificity of 94.7%, PPV of 92.3%, NPV of 90.0% and accuracy of 90.9% (12 TP, 18 TN, 1 FP, 2 FN), making it a reliable measure for confirming infection. Given its optimal balance of sensitivity and specificity, fracture SUV_peak_ with a cut-off value of 4.40 SUV is the preferred parameter for clinical use. An illustration can be found in Fig. 1, and an overview of all ROC-curve analyses is given in *Supplementary Table 2*.

### Dynamic scan

**Fig. 2:**
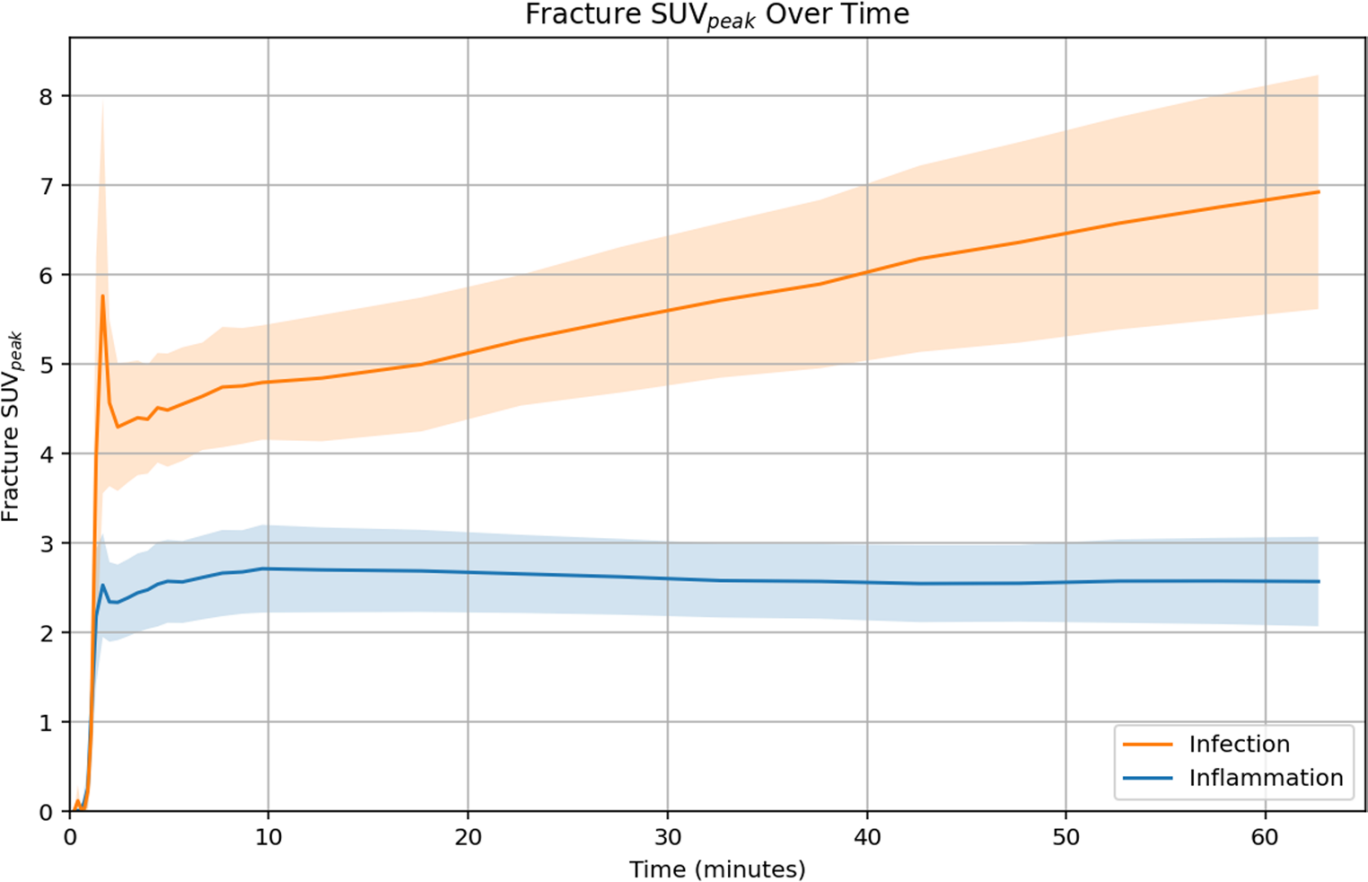
Fracture SUV_peak_ over time for inflammation and infection. The orange line represents the mean values for patients with confirmed bacterial infection, demonstrating a gradual increase over time, while the blue line represents the mean values for inflammation, remaining relatively stable. Shaded regions indicate the 95% confidence intervals. Abbreviations: SUV = standardized uptake value

Initial tracer uptake reached higher SUV_peak_ in infection compared to inflammation. After t = 10 min, infection cases showed a continuous increase in SUV_peak_, whereas inflammation cases displayed a flattening curve, indicating no significant change in tracer accumulation over time. The mixed-effects model provided a significant difference between the infection and inflammation group in the rate of change in fracture SUV_peak_ over time (*p* < 0.001 for the interactions), with the infection group showing a steeper increase in SUV_peak_ over time (Fig. 2). Other SUV measurements and their ratios can be found in *Supplementary Fig. 2*.

**Fig. 3:**
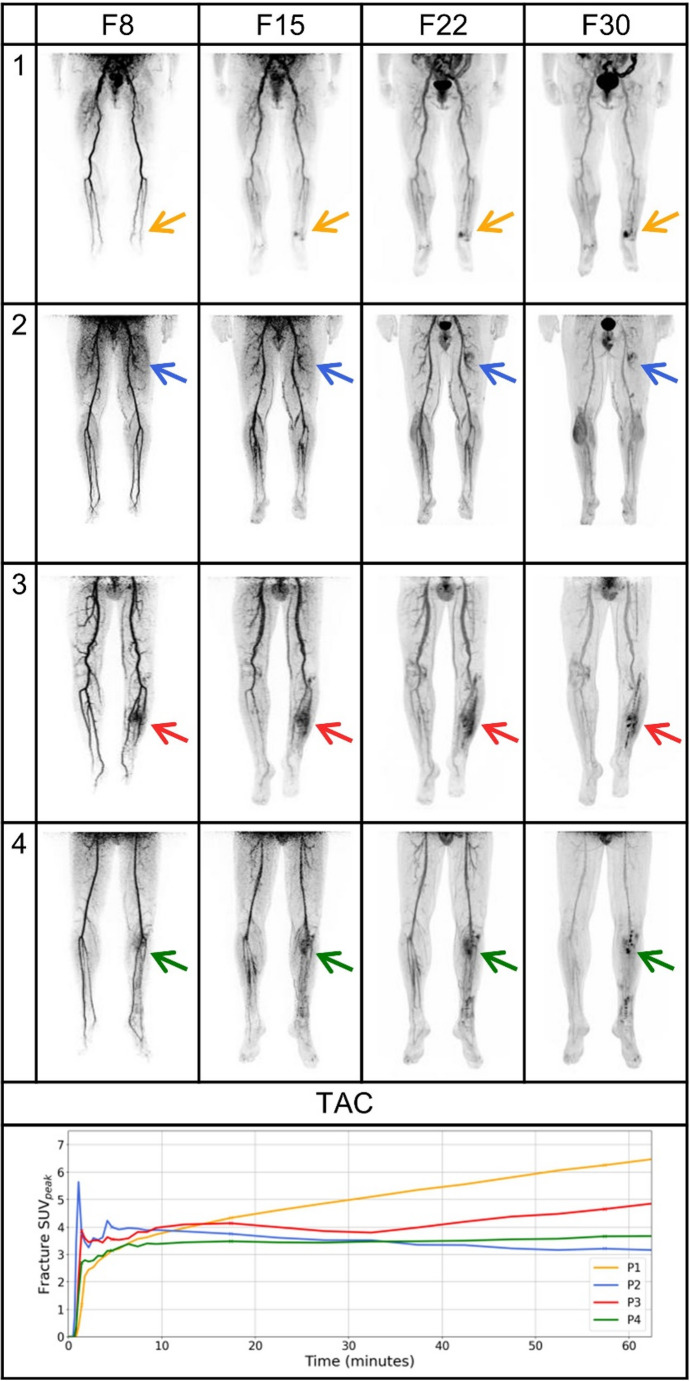
Dynamic [^18^F]FDG PET imaging in fracture-related infection assessment using time-activity curves (TACs). Frames 8 (80–100 s), 15 (4.5–5 min), 22 (15–20 min) and 30 (55–60 min) are shown for four patients. Patient 1 (true positive dynamic scan): a confirmed infected non-union of the medial malleolus showing progressive [^18^F]FDG uptake at the fracture site over time, with increasing SUV_peak_ values indicating infection. Patient 2 (true negative dynamic scan): a femoral non-union shows a visual and quantitative decrease in [^18^F]FDG uptake over time, confirming the abscense of infection. Patient 3 (true negative dynamic scan, whereas false positive on standard scan): a tibial non-union that is correctly classified as true negative based on visual and quantitative dynamic imaging, but was falsly classified as infection based on both visual and quantitative standard imaging. The visual [^18^F]FDG uptake around the fracture site seems only to delineate better over time, but does not seem to be progressive. The latter part of the curve shows actually SUV_peak_ progression, but it also reveals a ‘dip’ between 20 and 40 min, which may have caused a correction by TAC analysis and therefore the correct classification as inflammation. Patient 4 (false negative dynamic scan): a tibial non-union case of a false negative diagnosis by dynamic imaging, showing a flat uptake curve over time and no visual progression. This case was also falsly classified as negative for infection by quantitative standard imaging (SUV_peak_ < 4.40), but was correctly diagnosed as infection by visual standard assessment. Abbreviations: TAC = time-activity curve, SUV = standardized uptake value

Linear regression after t = 10 min showed the best fitting curves with Fracture SUV_peak_ = 0.0428 * time (minutes) + 4.2932 for the infection group and fracture SUV_peak_ = −0.0028 * time (minutes) + 2.6999 for the inflammation group. Comparing clinical diagnosis to each equation using the MSE, provided 12 true positives, 19 true negatives, 0 false positive and 2 false negatives, leading to a high sensitivity of 85.7%, a high specificity of 100%, PPV of 100% and NPV of 90.5%. The diagnostic accuracy is 93.9%. An illustration of specific patient cases can be found in Fig. 3.

When combining both visual standard analysis and quantitative TAC analysis, false positives from the visual analysis were reduced, while false negatives from the TAC method remained unchanged. Consequently, the overall diagnostic performance metrics were similar to those of the quantitative standard imaging, resulting in a diagnostic accuracy of 91%.

## Discussion

While dynamic [^18^F]FDG PET imaging has been explored in research settings, this study represents, to our knowledge, the first clinical study to distinguish bacterial infection from inflammation using time-activity curves as a standalone method. Our results suggest the potential of dynamic imaging to distinguish infection from inflammation with high diagnostic accuracy. Visual analysis alone achieved a diagnostic accuracy of 88%, while quantitative analysis using time-activity curves (TACs) further improved accuracy to 94%. Dynamic [^18^F]FDG imaging appears capable of capturing the rapid, sustained uptake seen in infections, driven by ongoing immune and microbial activity, in contrast to the more stable or plateau-like uptake pattern observed in inflammation. These initial results indicate that dynamic PET imaging could offer added value over conventional standard imaging, supporting our hypothesis that dynamic imaging can aid in differentiating infection from inflammation.

The proof-of-concept nature of this study confirms that dynamic imaging provides a quantifiable distinction between infectious and inflammatory processes. Notably, false positives were observed in visual analysis, which is in line with previous findings where [^18^F]FDG PET/CT imaging overestimates infection due to the overlap in [^18^F]FDG uptake with inflammatory conditions. In contrast, dynamic analysis reduced the false positive rate but introduced some false negatives. This shifts the diagnostic challenge, as false positives could lead to unnecessary interventions or treatments, while false negatives may result in missed infections and delayed clinical responses.

Standard quantitative analysis resulted in an overall diagnostic accuracy of 91%, demonstrating a slight improvement over the visual scoring of standard imaging (88%). Multiple studies have been performed on the diagnostic accuracy of [^18^F]FDG PET/CT imaging. For example, a retrospective study performed by Lemans et al. reported a sensitivity, specificity and diagnostic accuracy of 89%, 80% and 83%, respectively for diagnosis of FRI based on standard scan quantification [11]. Similarly, Bosch et al. found 76% sensitivity, 71% specificity, and 73% accuracy using conventional clinical standard image assessment [12], while van der Broeck et al. reported a sensitivity, specificity, and diagnostic accuracy of 67% in the context of low-grade infections [13]. A broader meta-analysis by Zhang et al. reported a sensitivity of 89% and specificity of 78% for [^18^F]FDG PET/CT [14]. Compared to these studies, our study shows a slightly higher diagnostic accuracy, which could be influenced by technical advancements enabled by a LAFOV PET/CT system. Dynamic imaging achieved a diagnostic accuracy of 94%, representing the highest performance among the evaluated approaches in this study. However, further validation in larger patient cohorts is needed to confirm these findings and establish the precise added value of dynamic imaging.

Despite its promising results, dynamic PET imaging presents several challenges. Patient movement remains a concern, as even small shifts can introduce motion artifacts and affect quantitative measurements. This challenge is particularly significant in low-grade infections or infections with biofilm formation, where subtle metabolic differences may be more difficult to detect. In this study, patients were positioned in a comfortable supine position with cushioning support to aid comfort, but no restraints were used, allowing for potential movement during the scan. Despite this, the high diagnostic accuracy achieved in our study suggests that dynamic PET imaging remains valuable even when patient movement is possible. Another challenge involves metal artifacts in the CT used for attenuation correction, which can affect PET quantification. While metal artifact reduction algorithms can mitigate these effects, such as the iterative metal artifact reduction (iMAR) used in this study, some distortion may persist. In this study, high diagnostic accuracy was achieved despite the presence of implants with the use of iMAR, suggesting limited impact under these conditions. Additionally, the one-hour acquisition time for dynamic imaging poses a logistical challenge, potentially impacting scan feasibility. However, this limitation may be addressed by optimizing the acquisition protocol. For example, reducing the scan duration to the interval of 40 to 60 min after injection instead of a full hour might be possible based on the TAC characteristics in this study and could improve feasibility in clinical practice.

This study has both strengths and limitations. As a proof-of-concept, it seems the first to demonstrate that dynamic PET imaging can distinguish infection from inflammation. The method’s simplicity—relying on TACs without the need for complex parametric mapping—is a strength in terms of clinical usability. However, this may also limit the depth of quantitative analysis. Additionally, the relatively small sample size (33 fractures, including 14 infections) and the specific population (patients with suspected FRI) restricts the generalizability of the findings, and especially side-by-side comparisons of imaging interpretation methods could not be performed. Although 11 out of 33 scans were rated as intermediate (10) or poor (1) quality due to patient movement during the extended dynamic acquisition leading to PET/CT misalignment, all scans remained clinically interpretable and were therefore included in the analysis. Despite these limitations, the encouraging results provide a solid foundation for further research and suggest a practical path toward clinical implementation.

A foundation for further research into dynamic [^18^F]FDG PET imaging for infection detection is provided by this study. Future studies should focus on larger patient cohorts and explore the applicability of dynamic imaging in other challenging infectious conditions, such as prosthetic joint infections, vascular graft infections, septic embolisms, and endocarditis. Additionally, investigating the role of dynamic PET imaging in early postoperative infections (< 3 months after surgery) could further define its clinical utility. By addressing these questions, this study aims to advance the use of non-invasive imaging techniques for improved infection diagnostics, with the potential to enhance patient management and treatment outcomes.

## Conclusion

This prospective study provides preliminary evidence that dynamic [^18^F]FDG PET/CT imaging using time-activity curve analysis could differentiate between bacterial infections and inflammation. Among the approaches for imaging evaluation, dynamic imaging using TACs achieved the highest diagnostic accuracy (94%) in fracture-related infections. Larger studies are needed to confirm these findings and determine the broader clinical value of dynamic [^18^F]FDG PET/CT imaging in routine clinical practice.

## Supplementary Information

Below is the link to the electronic supplementary material.

Supplementary Material 1 (DOCX. 671 KB)

## Data Availability

The anonymized datasets generated during and/or analyzed during the current study are available from the corresponding author on reasonable request.
