# Supplementary material for: Clinical evaluation of dynamic [18F]FDG PET imaging to distinguish infection from inflammation in fracture-related infections
Source: Eur J Nucl Med Mol Imaging. 2025 Sep 20;53(3):2167–77. doi: 10.1007/s00259-025-07563-x (PMC12860805; doi:10.1007/s00259-025-07563-x)
Supplement: Supplementary file 1 — Supplementary Material 1 (DOCX. 671 KB) [file 259_2025_7563_MOESM1_ESM.docx]

# Supplementary materials

|  | Infection | Inflammation | *p*-value |
| --- | --- | --- | --- |
| **SUV measurement** |  |  |  |
| Fracture SUV_max_ | 8.81 (6.99 – 10.62) | 3.44 (2.55 – 4.32) | **< 0.001** |
| Fracture SUV_peak_ | 6.97 (5.50 – 8.44) | 2.57 (2.02 – 3.11) | **< 0.001** |
| Contralateral SUV_peak_ | 1.67 (1.33 – 2.02) | 1.21 (1.04 – 1.38) | **0.018** |
| Femoral artery SUV_mean_ | 2.16 (1.61 – 2.72) | 1.68 (1.56 – 1.80) | 0.087 |
| **Ratio’s** |  |  |  |
| Fracture SUV_max_ / Femoral artery SUV_mean_ | 3.56 [3.09 – 5.86] | 1.78 [1.34 – 2.40] | **< 0.001** |
| Fracture SUV_peak_ / Femoral artery SUV_mean_ | 2.77 [2.47 – 4.27] | 1.52 (1.21 – 1.83) | **< 0.001** |
| Fracture SUV_max_ / Contralateral SUV_peak_ | 5.52 (3.86 – 7.17) | 2.44 [1.88 – 3.75] | **0.002** |
| Fracture SUV_peak_ / Contralateral SUV_peak_ | 4.37 (3.06 – 5.67) | 2.09 (1.68 – 2.49) | **0.003** |

*Supplementary Table 1: Quantitative measurements on standard [^18^F]FDG PET/CT imaging for fracture-related infections and their comparison. Data are presented as mean (95% CI) or as median [Q1-Q3].*

|  | AUC | Cut-off | Sensitivity | Specificity | PPV | NPV | Accuracy |
| --- | --- | --- | --- | --- | --- | --- | --- |
| **SUV measurement** |  |  |  |  |  |  |  |
| Fracture SUV_max_ | 0.947 | 5.32 | 0.929 | 0.894 | 0.867 | 0.944 | 0.909 |
| Fracture SUV_peak_ | 0.962 | 4.40 | 0.857 | 0.947 | 0.923 | 0.900 | 0.909 |
| Contralateral SUV_peak_ | 0.738 | 1.52 | 0.583 | 0.833 | 0.700 | 0.750 | 0.733 |
| **Ratio’s** |  |  |  |  |  |  |  |
| Fracture SUV_max_ / Femoral Artery SUV_mean_ | 0.923 | 3.03 | 0.846 | 0.895 | 0.846 | 0.895 | 0.875 |
| Fracture SUV_peak_ / Femoral Artery SUV_mean_ | 0.931 | 2.28 | 0.846 | 0.895 | 0.846 | 0.895 | 0.875 |
| Fracture SUV_max_ / Contralateral SUV_peak_ | 0.847 | 4.80 | 0.667 | 0.944 | 0.889 | 0.810 | 0.833 |
| Fracture SUV_peak_ / Contralateral SUV_peak_ | 0.875 | 3.91 | 0.667 | 1.000 | 1.000 | 0.818 | 0.867 |

*Supplementary Table 2: ROC-curve analysis based on quantitative measurements on standard [^18^F]FDG PET/CT imaging for fracture-related infections.*


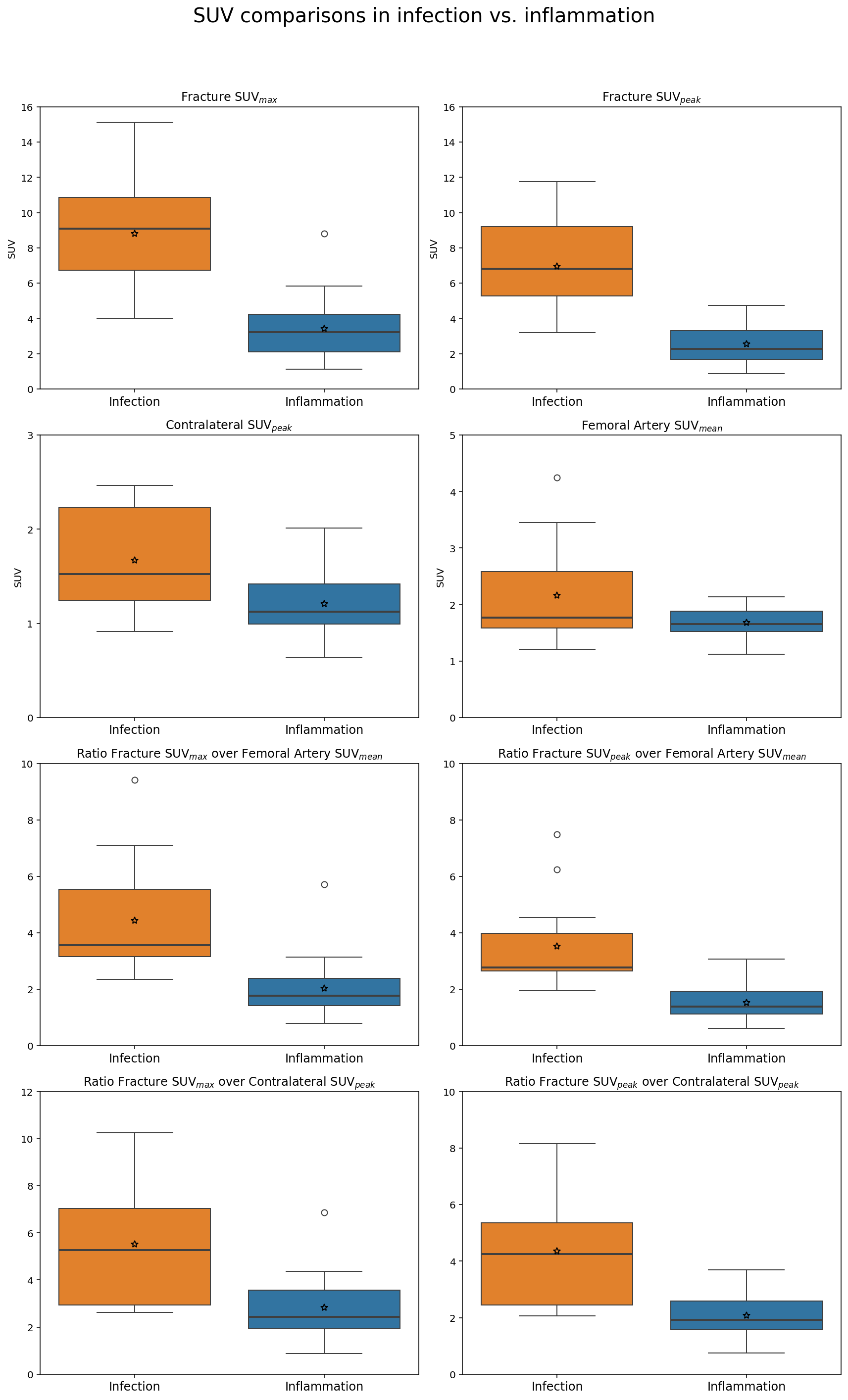


*Supplementary Figure 1:* *Visualized comparison of quantitative measurements on standard [^18^F]FDG PET/CT imaging for fracture-related infection using boxplots. The boxes visualize the inter-quartile range [Q1-Q3], the median and 1.5*IQR (whiskers). Outliers are presented with open dots. Mean values are showed by the asterisk. Abbreviations: SUV = standardized uptake value.*


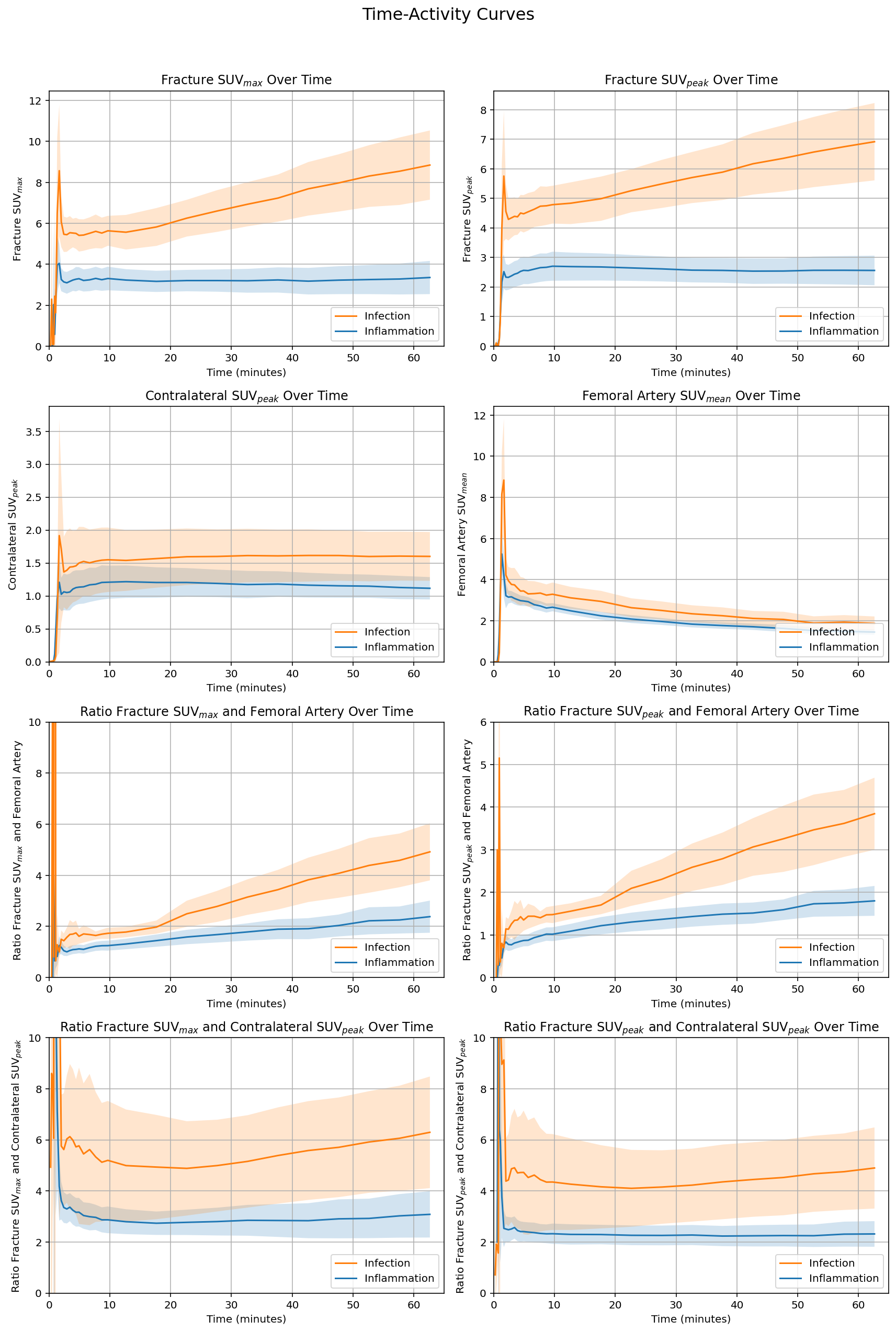


*Supplementary Figure 2: Fracture SUV_max_, Fracture SUV_peak_, contralateral SUV_peak_ and femoral artery SUV_mean_ and the ratios’s of fracture SUV_max_ and SUV_peak_ over femoral artery SUV_mean_ and contralateral SUV_peak_ over time for inflammation and infection. The orange line represents the mean values for infection, showing a progressive increase over time, while the blue line represents the mean values for inflammation, showing a slower increase over time. Shaded regions indicate the 95% confidence intervals. Abbreviations: SUV = standardized uptake value.*
